# Playfair’s System of Midwifery

**Published:** 1878-12

**Authors:** 


*Playfair's System of Midwifery.* A Treatise on the *Science* and *Prctice* of *Midwifery.* By W. S. Playfair, M. D., F. R. C. P. With Notes and Additions by Robert P. Harris, M. D. Second American from the second and Revised London Edition, with two plates and one hundred and eighty-two illustrations. Philadelphia: Henry C. Lea, 1878. T. H. Butler, Buffalo.

In this book we are furnished with a Text Book in Obstetrics, in which progress in midwifery is fully embodied. The author says his "object has been to place in the hands of his readers an epitome of the science and practice of midwifery which embodies all recent advances." The arrangement of this work is most excellent as a text book. A wide range of subjects are ably, clearly and succinctly discussed; the practical part of the various diseases is prominent in the discussion, so that it is a most useful guide in obstetrical practice. The work is very finely illustrated by wood cuts, some of which are copied from previous authors; some original.

In looking over the interesting and attractive chapters, we fall upon "The Transfusion of Blood," and make brief extracts from it as showing the plain, direct style of the author as well as his guarded and safe method of expression.

"The transfusion of blood in desperate and apparently hopeless cases of hemorrhage, offers a possible means of rescuing the patient which merits careful consideration.

"The *history of the operation* is of considerable interest. In Villari's "Life of Savonarola" it is said to have been employed in the case of Pope Innocent VIII., in the year 1492, but I am not aware on what authority the statement is made. The first serious proposals for its performance do not seem to have been made until the latter half of the seventeenth century. It was first actually performed tn France, by Dennis, of Montpellier, although Lower, of Oxford, had previously made experiments on animals which satisfied him that it might be undertaken with success. In November, 1667, some months after Denis's case, he made a public experiment at Arundel House, in which twelve ounces of sheep's blood were injected into the veins of a healthy man, who is stated to have been very well after the operation, which must, therefore, have proved successful. These nearly simultaneous cases give rise to a controversy as to priority of invention, which was long carried on with much bitterness.

The idea of resorting to transfusion after severe hemorrhage does not seem to have been then entertained. It was recommended as a means of treatment in various diseased states, or with the extravagant hope of imparting new life and vigor to the old and decrepit. The blood of the lower animals only was used; and, under these circumstances, it is not surprising that the operation, although practiced on several occasions, was never established as it might have been had its indications been better understood.

From that time it fell almost entirely into oblivion, although experiments and suggestions as to its applicability were occasionally made, especially by Dr. Harwood, Professor of Anatomy at Cambridge, who published a thesis on the subject in the year 1785. He, however, never carried his suggestions into practice, and, like his predecessors, only proposed to employ blood taken from the lower animals. In the year 1824 Dr. Blundell published his well known work, entitled "Researches, Physiological and Pathological," which detailed a large number of experiments; and to that distinguished physician belongs the undoubted merit of having brought the subject prominently before the profession, and of pointing out the cases in which the operation might be performed with hopes of success. Since the publication of this work, transfusion has been regarded as a legitimate operation under special circumstances; but, although it has frequently been performed with success, and in spite of many interesting monographs on the subject, it has never become so established, as a general resource in suitable cases, as its advantages would seem to warrant. Within the last few years more attention has been paid to the subject, and the writings of Panum, Martin, and de Belina, abroad, and of Higginson, McDonnell Hicks, and Aveling, at home, amongst many others, have thrown much light on many points connected with the operation, and it is to be hoped that the committee appointed by the Obstetrical Society, in their forthcoming report, may still more increase our knowledge.

